# Association between hospital and ICU structural factors and patient outcomes in China: a secondary analysis of the National Clinical Improvement System Data in 2019

**DOI:** 10.1186/s13054-022-03892-7

**Published:** 2022-01-21

**Authors:** Zhen Li, Xudong Ma, Sifa Gao, Qi Li, Hongbo Luo, Jianhua Sun, Wei Du, Longxiang Su, Lu Wang, Qing Zhang, Zunzhu Li, Xiang Zhou, Dawei Liu, China National Critical Care Quality Control Center Group, Xue Wang, Xiangdong Guan, Yan Kang, Bin Xiong, Bingyu Qin, Kejian Qian, Chunting Wang, Mingyan Zhao, Xiaochun Ma, Xiangyou Yu, Jiandong Lin, Aijun Pan, Haibo Qiu, Feng Shen, Shusheng Li, Yuhang Ai, Xiaohong Xie, Jing Yan, Weidong Wu, Meili Duan, Linjun Wan, Xiaojun Yang, Jian Liu, Hang Xu, Dongpo Jiang, Lei Xu, Zhuang Chen, Guoying Lin, Zhengping Yang, Zhenjie Hu

**Affiliations:** 1 Department of Critical Care Medicine, Peking Union Medical College Hospital, Peking Union Medical College and Chinese Academy of Medical Sciences, Beijing, 100730 China; 2 Department of Medical Administration, National Health Commission of the People’s Republic of China, Beijing, 100044 China

**Keywords:** ICUs, Critical care medicine, Staffing, Structure factors, Patient outcome, China

## Abstract

**Background:**

Hospital and ICU structural factors are key factors affecting the quality of care as well as ICU patient outcomes. However, the data from China are scarce. This study was designed to investigate how differences in patient outcomes are associated with differences in hospital and ICU structure variables in China throughout 2019.

**Methods:**

This was a multicenter observational study. Data from a total of 2820 hospitals were collected using the National Clinical Improvement System Data that reports ICU information in China. Data collection consisted of a) information on the hospital and ICU structural factors, including the hospital type, number of beds, staffing, among others, and b) ICU patient outcomes, including the mortality rate as well as the incidence of ventilator-associated pneumonia (VAP), catheter-related bloodstream infections (CRBSIs), and catheter-associated urinary tract infections (CAUTIs). Generalized linear mixed models were used to analyse the association between hospital and ICU structural factors and patient outcomes.

**Results:**

The median ICU patient mortality was 8.02% (3.78%, 14.35%), and the incidences of VAP, CRBSI, and CAUTI were 5.58 (1.55, 11.67) per 1000 ventilator days, 0.63 (0, 2.01) per 1000 catheter days, and 1.42 (0.37, 3.40) per 1000 catheter days, respectively. Mortality was significantly lower in public hospitals (*β* =  − 0.018 (− 0.031, − 0.005), *p* = 0.006), hospitals with an ICU-to-hospital bed percentage of more than 2% (*β* =  − 0.027 (− 0.034, -0.019), *p* < 0.001) and higher in hospitals with a bed-to-nurse ratio of more than 0.5:1 (*β* = 0.009 (0.001, 0.017), *p* = 0.027). The incidence of VAP was lower in public hospitals (*β* =  − 0.036 (− 0.054, − 0.018), *p* < 0.001). The incidence of CRBSIs was lower in public hospitals (*β* =  − 0.008 (− 0.014, − 0.002), *p* = 0.011) and higher in secondary hospitals (*β* = 0.005 (0.001, 0.009), *p* = 0.010), while the incidence of CAUTIs was higher in secondary hospitals (*β* = 0.010 (0.002, 0.018), *p* = 0.015).

**Conclusion:**

This study highlights the association between specific ICU structural factors and patient outcomes. Modifying structural factors is a potential opportunity that could improve patient outcomes in ICUs.

**Supplementary Information:**

The online version contains supplementary material available at 10.1186/s13054-022-03892-7.

## Background

Healthcare delivery is challenging and complex in the intensive care unit. Several factors, including ICU structure, ICU organization and the care process, can influence ICU performance [1–3]. Despite the extensive literature addressing the quality of care in ICUs, the impact of such factors remains controversial, and a diligent assessment of care components is required.

It is well known that ICU structural factors vary within different countries and regions [4, 5]. However, most related studies have been conducted in Western countries [6, 7]. Little evidence regarding the structural factors of ICUs in China is available [8]. China still faces challenges in providing optimal and equitable management strategies for ICU patients across the nation because of its broad geography and unbalanced economic development. A previous study described critical care resources in Guangdong Province [9]. Another study evaluated the practices, outcomes, and costs related to mechanical ventilation within ICUs in Beijing [10]. Nonetheless, those studies were limited to the regions in which the surveys were administered and feature small sample sizes; it is also not clear whether those resources were associated with ICU care provision, treatment patterns, and patient outcomes. Finally, increasing demands and rising costs pose significant challenges to the delivery of high-quality and affordable critical care to a growing population of patients. Optimizing ICU organization is a potential opportunity to improve patient outcomes and the use of resources.

Therefore, the aim of this study was to investigate hospital and ICU structural factors and patient outcomes in China. Moreover, we aimed to identify the association between these variables and patient outcomes, with a focus on potential structural factors, including ICU structural resources and staffing levels. We hypothesized that patients admitted to hospitals that were government-funded, tertiary, well-equipped, and better-staffed would have a decreased risk of ICU mortality and occurrence of VAP, CRBSIs and CAUTIs after adjusting for region, disease severity, and other confounders.

## Methods

### Design

This was a nationwide, observational database study in 2019. The data source was the National Clinical Improvement System (https://ncisdc.medidata.cn/login.jsp), collected by the China-National Critical Care Quality Control Centre (China-NCCQC), which is the official national department that regulates ICU quality control in China. The Ministry of Health of China approved that Peking Union Medical College Hospital establishes China-NCCQC in 2012. The Quality Improvement of Critical Care Program, led by China-NCCQC, was initiated in 2015. This study is part of the above program. Permission to use the data was obtained from the China-NCCQC.

### Study population and settings

The China-NCCQC collected the relevant data regarding quality control indicators through the database of the National Clinical Improvement System. Hospitals in China are classified in a 3-tier system (primary, secondary or tertiary hospital) that recognizes a hospital's ability to provide medical care, medical education, and conduct medical research. Tertiary hospitals, similar to a tertiary referral hospital in the West, are usually comprehensive, referral, general hospitals responsible for providing specialist health services, perform a larger role with regard to medical education and scientific research and serve as medical hubs providing care to multiple regions. Secondary hospitals, similar to a regional hospital or district hospital in the West, tend to be affiliated with a medium-sized city, county, or district and are responsible for providing comprehensive health services and medical education and conducting research on a regional basis. In contrast, primary hospitals are primary health care institutions whose main function is to provide primary prevention directly to the population, however, they rarely admit and treat critically ill patients. Therefore, primary hospitals were not included in the scope of the study.

The enrolled tertiary and secondary hospitals voluntarily participated and were selected by the China-NCCQC. The selection criteria were as follows. (1) The ICU had to have more than five beds. (2) The ICU had to have the ability to diagnose and treat the relevant medical diseases that were evaluated as quality control items (such as ventilator-associated pneumonia (VAP), catheter-related blood stream infections (CRBSIs), and catheter-associated urinary tract infections (CAUTIs)). Hospitals without ICUs were excluded from the study. The 31 provinces/municipalities/autonomous regions of mainland China were included in this survey (data from Hong Kong, Taiwan, and Macao were not included). There were 12,436 registered hospitals (including 2749 tertiary hospitals and 9687 secondary hospitals) across the country in 2019 [11], and a total of 2820 hospitals (including 1383 tertiary hospitals and 1437 secondary hospitals) in China were involved in the current analysis.

### Variables and measurements

#### Hospital and ICU structure factors

In this study, the structural factors of the hospital and ICU were evaluated according to the National Clinical Quality Control Indicators for Critical Care Medicine (2015 Edition) released by the China-NCCQC[12]. The structural indicators that were monitored included hospital characteristics and ICU characteristics in 2019. The hospital characteristics included the region (Eastern China, Central China, Western China, North-eastern China), location (metropolitan cities, other cities and rural areas), type (secondary, tertiary), ownership (private, public), and ICU-hospital bed percentages (calculated by the number of total ICU beds divided by the number of beds in the hospitals). The ICU characteristics included the physician-to-bed ratio (calculated by the total number of ICU physicians divided by the total number of ICU beds), bed-to-nurse ratio (calculated by the total number of ICU beds divided by the total number of full-time equivalent registered nurses working in the ICU), single rooms, and extracorporeal membrane oxygenation (ECMO) equipment. The proportion of ICU patients with APACHE II scores ≥ 15 (%) and the 6-h compliance rate with the surviving sepsis campaign guidelines (1. Completion of repeated measurement of lactate levels in patients with initial hyperlactatemia, 2. completion of resuscitation with vasopressors in patients with mean arterial pressure [MAP] ≤ 65 mmHg after fluid resuscitation, 3. completion of central venous pressure [CVP] and central venous oxygen saturation [ScvO2] measured in patients with lactate ≥ 4 mmol/L) and the microbiology detection rate before antibiotic use (defined as (no. of patients with microbiology detection before antibiotics)/(no. of patients who received antibiotics during the same period)) were also collected as controlling factors.

#### ICU patient outcomes

The ICU patient outcomes included the ICU mortality rate and the incidence of VAP, CRBSIs and CAUTIs in 2019. The ICU mortality rate (%) was defined as the number of patients who died in the ICU/the number of patients admitted to the ICU during the same period. The VAP incidence rate per 1000 ventilator days was defined as the number of patients with VAP/the number of patients with mechanical ventilation during the same period. The CRBSI incidence rate per 1000 catheter days was defined as the number of patients with CRBSIs/the number of patients with a central venous catheter during the same period. The CAUTI incidence rate per 1000 catheter days was defined as the number of patients with CAUTIs/the number of patients with a urinary catheter during the same period [13]. The definitions of these outcome indicators are described in Table S1 in Additional file 1.

### Data collection

The data were collected between January 1, 2019, and December 31, 2019, and were entered into a web-based data entry system by a local, trained independent research coordinator. Range checks were used to check for inconsistent or out-of-range data, prompting the user to correct or review data entries outside the predefined range. The system also used predefined logical checks to identify any errors or illogical data entries. A data quality meeting was held monthly to review all hospital enrolment records and registry data.

### Ethical considerations

The current study is reported in accordance with the Strengthening the Reporting of Observational Studies in Epidemiology guidelines. The study was conducted in accordance with the Declaration of Helsinki (as revised in 2013). The trial protocol was approved by the Central Institutional Review Board at Peking Union Medical College Hospital (NO. SK1828), and individual consent for this analysis was waived. No identifying or protected health information was included in the analysed dataset.

### Data analysis

All statistical analyses were performed in SAS 9.4 (SAS Institute Inc., Cary, NC, USA). Normally distributed data are expressed as the mean and standard deviation and were compared using Student’s t test. Nonnormally distributed data are presented as the median and interquartile range (IQR) and were analysed using the nonparametric Mann–Whitney U test. To identify the adjusted effects of the structural variables on patient outcomes, a multivariate analysis was conducted using generalized linear mixed models with two random intercept models to demonstrate the effects of the region and location. The model took into account the fact that patients from the same region or city may have unmeasured characteristics that are similar because the patient outcomes would be more similar in patients from the same region than in patients that are across different regions. The mixed-effects model analyses were run separately for each patient outcome. Covariates that were considered to be important impact factors in the patient outcomes based on the prior literature and the univariate analysis were taken as candidates for inclusion in the models. In addition, patient variables (the proportion of ICU patients with an APACHE II score ≥ 15 (%)) and processing factors (the 6-h SSC bundle compliance rate (%) and the microbiology detection rate before antibiotic use (%)) were also included in the model. The results are expressed as the *p* value and beta (*β*) with the 95% confidence interval (CI). A missing value analysis was conducted. The percentage of missing values across the variables varied between 0 and 12.02%. In total, 2387 observations were complete (84.65%). Listwise deletion was used to handle missing data. All statistical tests were two-tailed, and *p* < 0.05 was considered to be statistically significant.

## Results

### Hospital structure characteristics

A total of 2820 hospitals from 31 provinces were included in the data analysis. All hospital structural characteristics were analysed and are presented in Table 1. The number of tertiary hospitals was 1383 (49.04%), compared to 1437 (50.96%) secondary hospitals. A larger proportion of the hospitals were from Western China (1083, 38.40%), were public (2574, 91.28%), and were located in nonmetropolitan cities or rural areas (2288, 81.13%). The median ICU-hospital bed percentage was 1.77% (1.29%, 2.42%). There were significant differences in all hospital characteristics between the tertiary and secondary hospitals, except for the ICU-hospital bed percentage.

**Table 1:** Characteristics of the hospitals

Variables	Categories	Total *n*(%)	Tertiary hospital *n*(%)	Secondary hospital *n*(%)	*X*^2^	*p*
Region	Eastern China	889(31.52)	399(28.85)	490(34.10)	84.441	< 0.001
	Central China	659(23.37)	270(19.52)	389(27.07)		
	Western China	1083(38.40)	570(41.21)	513(35.70)		
	North-eastern China	189(6.70)	144(10.41)	45(3.13)		
Location	Metropolitan cities	532(18.87)	353(25.52)	179(12.46)	77.773	< 0.001
	Other cities and rural areas	2288(81.13)	1030(74.48)	1258(87.54)		
Hospital ownership	Private	246(8.72)	80(5.78)	166(11.55)	29.440	< 0.001
	Public	2574(91.28)	1303(94.22)	1271(88.45)		
Number of beds in the hospital	Median(Q1,Q3)	730(491,1195)	1124 (7621672	506(360,707)		
	< 500	773(27.41)	102(7.38)	671(46.69)	960.486	< 0.001
	500–1000	1181(41.88)	512(37.02)	669(46.56)		
	> 1000	866(30.71)	769(55.60)	97(6.75)		
Number of beds in the ICU	Median(Q1,Q3)	12(8,20)	18(12,30)	9(6,12)		
	< 10	958(33.97)	218(15.76)	740(51.50)	654.300	< 0.001
	10–15	842(29.86)	356(25.74)	486(33.82)		
	> 15	1020(36.17)	809(58.50)	211(14.68)		
ICU: hospital bed percentage (%)						
	< 2%	1708(60.57)	855(61.82)	853(59.36)	1.789	0.181
	≥ 2%	1112(39.43)	528(38.18)	584(40.64)		

### ICU structure characteristics

All ICU structural characteristics are presented in Table 2. The median physician-to-bed ratio was 0.60 (0.44, 0.78), while the median bed-to-nurse ratio was 0.55 (0.44, 0.71). A large proportion (59.57%) of the ICUs had more than one private patient room. Respondents reported 450 ICUs equipped with ECMO (15.96%). The median proportion of patients with an APACHE II score ≥ 15 24 h after admission was 58.91% (35.12%, 76.30%). The median proportion of the 6 h SSC bundle compliance rate (%) was 80.00% (50.00%, 100%), while the median proportion of the microbiology detection rate before antibiotic use (%) was 91.67% (71.47%, 100%). Moreover, there were significant differences in all of these characteristics between the tertiary and secondary hospitals.

**Table 2:** Characteristics of the ICUs

Variables	Categories	Total	Tertiary hospital	Secondary hospital	*X*^2^	*p*
Number of physicians	Median(Q1,Q3)	7(5,11)	10(7,17)	6(4,7)		
Physician-to-bed ratio	Median(Q1,Q3)	0.60(0.44,0.78)	0.58(0.44,0.75)	0.60(0.44,0.80)		
	< 0.6	1425(50.53)	730(52.78)	695(48.36)	5.330	0.0210
	≥ 0.6	1395(49.47)	653(47.22)	742(51.64)		
Number of nurses	Median(Q1,Q3)	21(14,36)	34(21,57)	16(11,22)		
Bed-to-nurse ratio	Median(Q1,Q3)	0.55(0.44, 0.71)	0.52(0.42, 0.68)	0.58(0.48,0.75)		
	< 0.5	1000(35.46)	590(42.66)	410(28.53)	60.858	< 0.001
	≥ 0.5	1820(64.54)	793(57.34)	1027(71.47)		
Single room in the ICU	Median(Q1,Q3)	2(1,4)	3(2,6)	1(1,2)		
	≤ 1	1140(40.43)	335(24.22)	805(56.02)	294.537	< 0.001
	> 1	1680(59.57)	1048(75.78)	632(43.98)		
ECMO	Median(Q1,Q3)	0(0,0)	0(0,1)	0(0,0)		
	0	2370(84.04)	956(69.13)	1414(98.40)	448.161	< 0.001
	≥ 1	450(15.96)	427(30.87)	23(1.60)		
Proportion of patient with an APACHEII score ≥ 15 in 24 h after admission (%)	Median(Q1,Q3)	58.91(35.12,76.30)	59.28(37.30,76.26)	58.06(33.33,76.58)		
	< 50%	1342(47.59)	619(44.76)	723(50.31)	8.499	0.003
	≥ 50	1478(52.41)	764(55.24)	714(49.69)		
6 h SSC bundle compliance rate (%)	Median(Q1,Q3)	80.00(50.00,100)	82.85(55.94,100)	75.00(42.86,100)		
	< 90%	1936(68.65)	722(52.21)	935(65.07)	47.572	< 0.001
	≥ 90%	884(31.35)	661(47.79)	502(34.93)		
Microbiology detection rate before antibiotic use(%)	Median(Q1,Q3)	91.67(71.47,100)	94.53(78.87,100)	88.14(63.44,100)		
	< 90%	1485(52.66)	622(44.97)	863(60.06)	63.692	< 0.001
	≥ 90%	1335(47.34)	761(55.03)	574(39.94)		

### ICU patient outcomes

The health outcomes of ICU patients were analysed and are presented in Table 3. Overall, the median ICU patient mortality was 8.02% (3.78%, 14.35%). In addition, the incidence of VAP (median: 5.58 (95%CI: 1.55, 11.67) per 1000 ventilator days) was higher than that of CRBSIs (median: 0.63 (95%CI: 0, 2.01) per 1000 catheter days) and CAUTIs (median: 1.42 (95%CI: 0.37, 3.40) per 1000 catheter days). There were significant differences in patient outcomes between the tertiary and secondary hospitals except for the incidence of VAP.

**Table 3:** ICU Patient health outcomes

Variables	*n*	Total median (Q1,Q3)	Tertiary hospital median (Q1,Q3)	Secondary hospital median (Q1,Q3)	*X*^2^	*p*
Mortality in the ICU (%)	2707	8.02(3.78, 14.35)	8.94(4.49, 15.23)	7.14(3.29, 13.33)	23.650	< 0.001
VAP (per 1000 ventilator days)	2546	5.58(1.55, 11.67)	5.63(2.20, 10.57)	5.44(0, 13.74)	2.805	0.094
CRBSIs (per 1000 catheter days)	2481	0.63(0, 2.01)	0.83(0, 1.98)	0(0, 2.06)	46.890	< 0.001
CAUTIs (per 1000 catheter days)	2548	1.42(0.37,3.40)	1.32(0.45, 2.77)	1.64(0, 4.44)	6.803	0.010

### Association between hospital and ICU structural factors and ICU patient outcomes

**Fig. 1:**
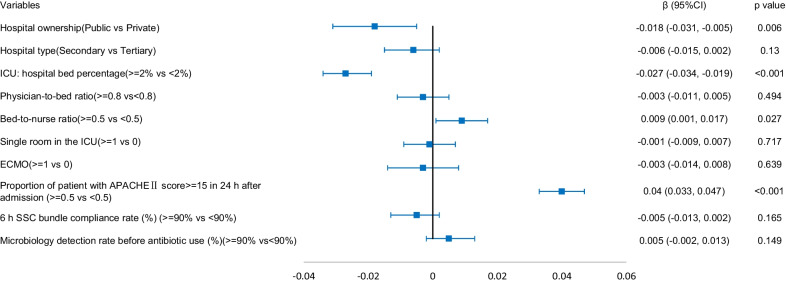
The adjusted effect of hospital and ICU structural factors on patient mortality based on generalized linear mixed models

**Fig. 2:**
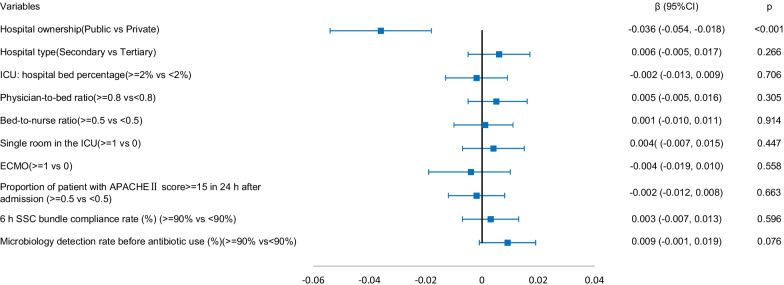
The adjusted effect of hospital and ICU structural factors on the incidence of VAP based on generalized linear mixed models

**Fig. 3:**
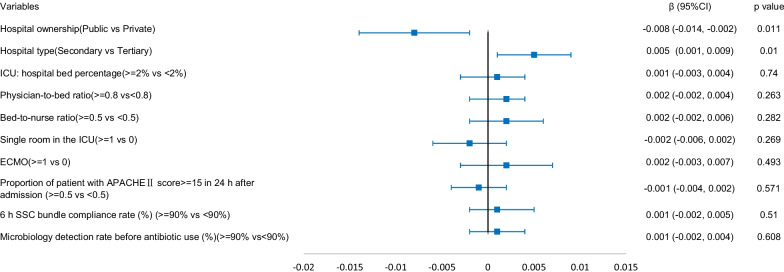
The adjusted effect of hospital and ICU structural factors on the incidence of CRBSIs based on generalized linear mixed models

**Fig. 4:**
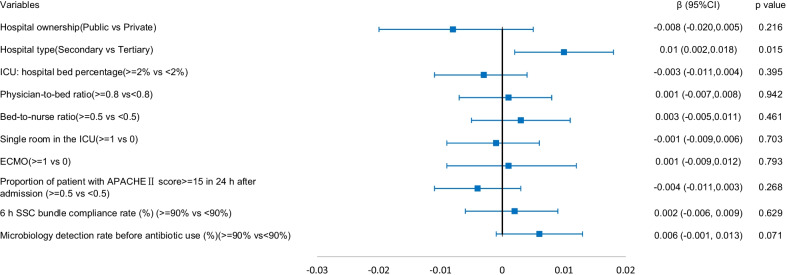
The adjusted effect of hospital and ICU structural factors on the incidence of CAUTIs based on generalized linear mixed models

The results of the generalized linear mixed model are shown in Figs. 1, 2, 3, 4. Structural factors associated with lower ICU patient mortality included public hospitals (*β* = -0.018 (-0.031, − 0.005), *p* = 0.006), hospitals with an ICU-to-hospital bed percentage of more than 2% (*β* = -0.027 (-0.034, -0.019), *p* < 0.001), and ICU patient mortality was higher in hospitals with a bed-to-nurse ratio of more than 0.5:1 (*β* = 0.009 (0.001, 0.017), *p* = 0.027). The incidence of VAP was lower in public hospitals (*β* = -0.036 (-0.054, − 0.018), *p* < 0.001), the incidence of CRBSIs was lower in public hospitals (*β* = -0.008 (-0.014, − 0.002), *p* = 0.011) and higher in secondary hospitals (*β* = 0.005 (0.001, 0.009), *p* = 0.010), and the incidence of CAUTIs was higher in secondary hospitals (*β* = 0.010 (0.002, 0.018), *p* = 0.015).

## Discussion

Gaps and variations in patient care and outcomes for ICU patients exist within and across countries worldwide, particularly between developed and developing regions. In this study, we report detailed information on the structural factors for a large sample of Chinese ICUs. We found that the hospital and ICU structure and patient outcomes varied substantially among the participating hospitals. Some of the structural factors were associated with ICU patient outcomes.

The population of China has aged rapidly, and the rate will continue to accelerate in the decades to come. Meanwhile, the number of hospitals and hospital volume is gradually increasing. An ageing society could also induce an increase in ICU admissions and ICU demand. Studies have shown that the organization, structure, and delivery of critical care in China are different from those in Asia, Europe and North America [13–16]. Critical care medicine in mainland China is still in a phase of development. China still has a large gap with developed countries in the number of ICU beds and capacity, clinician staffing, critical care technicians (such as respiratory therapists), ICU equipment, and so on. For example, the rapid expansion of hospital beds was disproportionate to the severe shortage of ICU beds. The ratio of ICUs to hospital beds recommended by the Guidelines for the Construction and Management of Critical Care Medicine in China was 2–8% [17]. Although the proportion is relatively lower than that in many developed countries, a large number of hospitals did not meet this recommendation [18]. Under the condition of limited and unevenly distributed ICU resources, exploring the impact of organizational and structural factors on ICU patient outcomes in Chinese ICUs could provide a valuable reference for the further improvement of critical care quality. To our knowledge, this is the first national report on hospital- and unit-level differences in medical care and outcomes for ICU patients in China, and it reveals the gaps and challenges that China is facing. These findings establish the fundamental and current status for the care and outcomes of ICU patients and serve as a basis to guide efforts for quality improvements in intensive care and in the allocation of resources.

Our results showed that hospital ownership was significantly associated with ICU patient outcomes. The mortality rate and incidence of VAP and CRBSIs in ICU patients admitted to private hospitals were higher than those in patients admitted to public hospitals. There are some possible explanations for this finding. Hospitals in China are classified into public hospitals and private hospitals according to ownership and economic type. Public hospitals are non-profit and receive financial subsidies from the state, so their medical prices are strictly limited. Private hospitals, which are believed to be indispensable supplements to public hospitals to enhance healthcare quality and efficiency across the nation and to meet the rapidly increasing demand for diversified health care, are generally profit-making hospitals under the government’s supervision and are responsible for their profits and losses, with independent decisions made on medical prices. Since 1980, private hospitals have begun to appear in China’s medical industry. The large-scale development of private hospitals in China occurred after 2001. The number of private hospitals exceeded that of public ones in 2015. In 2019, the number of private hospitals in China reached 22,424, while the number of public hospitals had declined to 11,930 [11]. Despite the growth in the number of facilities, private hospitals still face several challenges in the Chinese social and medical context. In a relatively short period of development, private hospitals are more likely to be smaller and specialized hospitals, and the most substantial issues facing private hospitals are the recruitment of high-quality physicians and the lack of public insurance coverage. While public hospitals typically have more beds, staff, and tertiary care capacity, they usually allocate sufficient medical talent, equipment and resources to meet the needs of patients [19]. A recent study in Beijing, China showed that the technical efficiency, pure technical efficiency, and scale efficiency of public hospitals were higher than those of private hospitals [20]. In addition, public hospitals usually have a better reputation than private hospitals, and reputation also influences the performance and efficiency of hospitals. As a result, differences exist in the outcomes of ICU patients in public and private hospitals. With the rapid development of private hospitals in China, measures need to be taken to further improve the quality of care and outcomes of ICU patients in private hospitals.

In this study, ICU patients admitted to tertiary hospitals in China had a lower CAUTI incidence than patients admitted to secondary hospitals. Being treated in a secondary hospital was associated with a higher CAUTI incidence in the multivariate analysis. The difference in the clinical care, diagnostic protocols, assessment, and treatment of hospital infection might account for the observed discrepancy in the CAUTI incidence among ICU patients. Recent studies have also observed higher mortality rates in secondary hospitals than in tertiary hospitals [21, 22]. These complications also reflect the combined effect of patient case-mix and quality of care. In China, tertiary hospitals are usually comprehensive, referral-based, general hospitals responsible for providing specialist health services and performing a larger role with regard to medical education and scientific research. They also serve as medical hubs providing care to multiple regions, while secondary hospitals are responsible for providing comprehensive health services, medical education and conducting research on a regional basis. For one thing, patients with acute and critical illnesses tend to be treated in tertiary hospitals rather than secondary hospitals, resulting in a higher proportion of patients with acute and critical illnesses in tertiary care hospitals, and for another, tertiary hospitals are usually more adequately staffed and equipped compared to secondary hospitals, which may lead to a lower rate of complications in ICU patients from tertiary hospitals. Intensive training and technical support for ICU staff in secondary hospitals should be implemented to narrow the gaps and variations in the care and outcomes of ICU patients.

We found that an ICU-to-hospital bed percentage of more than 2% was independently associated with a lower mortality rate in ICU patients, which is consistent with a contemporary study [23]. Previous studies indicated that larger hospitals and hospitals with high ICU occupancy were more likely to increase their number of ICU beds compared to other hospitals. Small hospitals and hospitals with relatively low ICU occupancy were less likely to add ICU beds in the subsequent year [24]. When the ICU occupancy rate is high, lower ICU bed ratios could possibly result in delayed ICU admissions, which in turn may affect patient outcomes. It is noteworthy that the result should be interpreted with caution. A combination of factors, including ICU bed occupancy rate, acuity of patients, and capacity of other departments (e.g., operation rooms), should be considered when making the decision whether to expand ICUs in a hospital.

A large number of studies have reported that a higher number of nursing staff was associated with a lower in-hospital mortality rate [7, 22, 25, 26]. Our study showed a similar trend. Patients from an ICU with a bed-to-nurse ratio of more than 0.5 (two nurses per bed) had significantly higher mortality. The bed-to-nurse ratio is a widely used indicator of nurse staffing in ICUs and general wards, and larger numbers of bed-to-nurses indicate worse staffing. ICU patients are highly dependent on nursing care due to the nature of their illnesses, the need for continuous invasive monitoring, and the need for multiple organ system support. Variables that mediated the relationship between nurse staffing and the patient outcome of death were inferred to be insufficient physician collaboration, excessive workload, increased medical errors, and missed nursing care [3, 27]. Moreover, one key role that ICU nurses perform is patient monitoring. ICU nurses are at the patient’s bedside around the clock and are paramount for the early identification of problems. Failure of such monitoring may cause life-threatening complications such as pneumothorax or unexpected extubation, which requires prompt recognition and treatment [28]. A shortage of nursing staff could be associated with insufficient supervision and might inhibit the early recognition of any changes in the status of the patients [25]. This finding could provide useful information for nurse managers and policymakers to determine if staffing levels are adequate and safe, not just whether there is a relationship between staffing and outcomes. This finding should be considered in light of the lower nurse-to-patient ratios in China and other low-and middle-income countries. It should also be noted that the average bed-to-nurse ratio used in this study would not be equal to the nurse-to-patient ratio of the units where the numbers of nurses and patients are in constant dynamic change. Furthermore, the quality of the nursing staff (education course, advanced training) could possibly be a confounder of ICU patient mortality. Future research that uses a more advanced study design and analytical approach is needed to examine the dynamic impact of nurse staffing on ICU patient outcomes.

There are some limitations to this study. First, the use of secondary data limited essential variables such as the patient-level characteristics or uncontrolled confounders (e.g., education level of the staff, ICU bed occupancy rate) needed to understand the predictive factors of the patients’ outcomes. To reduce these methodological problems, we applied multivariate analysis using generalized linear mixed models adjusted for patient disease severity using the APACHE II score, which has been shown to be an important predictor of patient outcomes [29, 30]. Second, since only one year of data was reported, presented and summarized in this study, the relationships of the structural factors and health outcomes could not be analysed continuously and dynamically. Third, this was an observational study and, therefore, prone to selection bias. Causal relationships cannot be drawn due to the cross-sectional nature of the study design. Fourth, listwise deletion was used to handle the missing data, which may cause bias in the estimates of the parameters [31]. Despite these limitations, the results of this study are highly meaningful in that they underscore the necessity of nonpatient factors, including hospital and ICU structural factors, as a way to reduce adverse patient outcomes. In addition, we have improved the generalizability of the findings by using national administrative data, unlike most previous studies, which were conducted using data from only certain regions or hospitals. The results may have important implications for critical care development in China and other countries with similar medical environments.

## Conclusion

In conclusion, specific structural factors, including hospital ownership, hospital type, ICU-hospital bed percentage, and bed-to-nurse ratio, were associated with ICU patient outcomes. These observations can assist in policies and interventions to bridge the current quality gap in the delivery of critical care in China as well as other developing countries.

## Supplementary Information

**Additional file 1.** Definition of ventilator-associated pneumonia, catheter-related bloodstream infections, and catheter-associated urinary tract infections.

## Data Availability

The datasets analysed during the current study are available from the corresponding author on reasonable request.

## Abbreviations

VAP: Ventilator-associated pneumonia
CRBSIs: Catheter-related blood stream infections
CAUTIs: Catheter-associated urinary tract infections
APACHE II score: Acute Physiology and Chronic Health Evaluation score
IQR: Interquartile range
95% CI: Confidence interval
ICU: Intensive care unit
