# Correction to: Association between hospital and ICU structural factors and patient outcomes in China: a secondary analysis of the National Clinical Improvement System Data in 2019

**DOI:** 10.1186/s13054-022-03920-6

**Published:** 2022-02-11

**Authors:** Zhen Li, Xudong Ma, Sifa Gao, Qi Li, Hongbo Luo, Jianhua Sun, Wei Du, Longxiang Su, Lu Wang, Qing Zhang, Zunzhu Li, Xiang Zhou, Dawei Liu, China National Critical Care Quality Control Center Group, Xue Wang, Xiangdong Guan, Yan Kang, Bin Xiong, Bingyu Qin, Kejian Qian, Chunting Wang, Mingyan Zhao, Xiaochun Ma, Xiangyou Yu, Jiandong Lin, Aijun Pan, Haibo Qiu, Feng Shen, Shusheng Li, Yuhang Ai, Xiaohong Xie, Jing Yan, Weidong Wu, Meili Duan, Linjun Wan, Xiaojun Yang, Jian Liu, Hang Xu, Dongpo Jiang, Lei Xu, Zhuang Chen, Guoying Lin, Zhengping Yang, Zhenjie Hu

**Affiliations:** 1 Department of Critical Care Medicine, Peking Union Medical College Hospital, Chinese Academy of Medical Sciences, Beijing, 100730 China; 2 Department of Medical Administration, National Health Commission of the People’s Republic of China, Beijing, 100044 China

## Correction to: Critical Care (2022) 26:24 10.1186/s13054-022-03892-7

Following publication of the original article [1], the authors identified an error in the corresponding authors. The author Zhen Li is first author not a corresponding author.

The author group has been updated above and the original article [1] has been corrected.

## References

[1] Li Z (2022). Association between hospital and ICU structural factors and patient outcomes in China: a secondary analysis of the National Clinical Improvement System Data in 2019. Criti Care.

