# Supplementary material for: Association between hospital and ICU structural factors and patient outcomes in China: a secondary analysis of the National Clinical Improvement System Data in 2019
Source: Crit Care. 2022 Jan 21;26:24. doi: 10.1186/s13054-022-03892-7 (PMC8780710; doi:10.1186/s13054-022-03892-7)
Supplement: Supplementary file 1 — Additional file 1. Definition of ventilator-associated pneumonia, catheter-related bloodstream infections, and catheter-associated urinary tract infections. [file 13054_2022_3892_MOESM1_ESM.docx]

Table S1 Definition of ventilator-associated pneumonia, catheter-related bloodstream infections, and catheter-associated urinary tract infections

| Category | Definition |
| --- | --- |
| Ventilator-associated pneumonia | VAP diagnosis relied on clinical, radiological, and microbiological criteria. Namely, new and persistent infiltrate on chest X-rays (CXR) was associated with two of the following criteria: (1) turbid tracheal aspirates, (2) temperature > 38 °C or < 36 °C, and (3) peripheral leukocyte count > 10 G/L or < 1.5 G/L). All VAP diagnoses were documented by a positive microbiological sample of tracheal aspirate (≥ 105 CFU/mL), protected telescopic catheter liquid (≥ 103 CFU/mL), or bronchoalveolar lavage (≥ 104 CFU/mL). Tracheobronchial colonization was confirmed by a positive (≥ 105 CFU/ml) tracheal aspirate without CXR signs of VAP. |
| Catheter-related blood stream infection | **Criteria 1:**The patient had a recognized pathogen cultured from ≥1 blood cultures (the term “recognized pathogen” does not include organisms considered common skin contaminants, i.e., those that can be cultured from ≥2 blood cultures drawn on separate occasions).  **Criteria 2**: The patient has one or more of the following signs/symptoms unrelated to infection at any other site.: fever ≥38.0 °C, chills, or hypotension.  **Criteria 3:** The same organism is obtained from ≥1 percutaneous blood culture as well as a catheter-tip culture. |
| Catheter-associated urinary tract infections | The presence of symptoms or signs compatible with UTI with no other identified source of infection along with ⩾103 colony-forming units (cfu)/mL of ⩾1 bacterial species in a single catheter urine specimen or in a midstream voided urine specimen from a patient whose urethral, suprapubic, or condom catheter has been removed within the previous 48 h. |
